# Prolonged Controlled Oxygenated Rewarming Improves Immediate Tubular Function and Energetic Recovery of Porcine Kidneys During Normothermic Machine Perfusion

**DOI:** 10.1097/TP.0000000000004427

**Published:** 2022-11-18

**Authors:** Baran Ogurlu, Carolina C. Pamplona, Isa M. Van Tricht, Tim L. Hamelink, Veerle A. Lantinga, Henri G.D. Leuvenink, Cyril Moers, Merel B.F. Pool

**Affiliations:** 1 Department of Surgery – Organ Donation and Transplantation, University Medical Center Groningen, Groningen, The Netherlands.

## Abstract

Normothermic machine perfusion (NMP) is typically performed after a period of hypothermic preservation, which exposes the kidney to an abrupt increase in temperature and intravascular pressure. The resultant rewarming injury could be alleviated by gradual rewarming using controlled oxygenated rewarming (COR). This study aimed to establish which rewarming rate during COR results in the best protective effect on renal rewarming injury during subsequent NMP.

**Methods.:**

Twenty-eight viable porcine kidneys (n = 7/group) were obtained from a slaughterhouse. After these kidneys had sustained 30 min of warm ischemia and 24 h of oxygenated HMP, they were either rewarmed abruptly from 4–8 °C to 37 °C by directly initiating NMP or gradually throughout 30, 60, or 120 min of COR (rate of increase in kidney temperature of 4.46%/min, 2.20%/min, or 1.10%/min) before NMP.

**Results.:**

Kidneys that were rewarmed during the course of 120 min (COR-120) had significantly lower fractional excretion of sodium and glucose at the start of NMP compared with rewarming durations of 30 min (COR-30) and 60 min (COR-60). Although COR-120 kidneys showed superior immediate tubular function at the start of normothermic perfusion, this difference disappeared during NMP. Furthermore, energetic recovery was significantly improved in COR-30 and COR-120 kidneys compared with abruptly rewarmed and COR-60 kidneys.

**Conclusions.:**

This study suggests that a rewarming rate of 1.10%/min during COR-120 could result in superior immediate tubular function and energetic recovery during NMP. Therefore, it may provide the best protective effect against rewarming injury.

## INTRODUCTION

Normothermic (35–37 °C) machine perfusion (NMP) has emerged as a promising platform that could enable the assessment of renal suitability for transplantation as it approaches physiological conditions.^1-3^ For the purpose of pretransplant renal viability assessment, a few hours (1–6 h) of NMP are typically implemented after a period of hypothermic preservation (in-house NMP).^4-7^ However, in-house NMP exposes the kidney to an abrupt increase in intravascular pressure and temperature, which induces rewarming injury. During NMP and after transplantation, additional injury occurs with the abrupt rewarming of the cold kidney. Although most of the underlying injurious processes remain to be elucidated, rewarming injury encompasses several important aspects of tissue damage, which are associated with impaired renal function during reperfusion. It has been postulated that altered mitochondrial integrity upon rewarming is the main culprit for posttransplant renal dysfunction.^8^ Ischemia and subsequent reperfusion induce increased production of reactive oxygen species (ROS) and the resultant damage to adjacent biomolecules (proteins, lipids, and DNA) eventually leads to cell death.^9,10^ Increased ROS production also stimulates the opening of mitochondrial transition pores. The associated loss of mitochondrial membrane potential leads to apoptosis, a process that is potentiated by abrupt rewarming.^11^ The swift increase in temperature also results in a decrease in the efficiency of oxygen utilization through mitochondrial uncoupling.^8^ Consequently, the mitochondria of the organ are unable to produce extra ATP via oxidative phosphorylation. Rewarming injury can be ameliorated by gradually increasing the renal temperature and perfusate pressure using controlled oxygenated rewarming (COR) as a thermal transition phase between hypothermic preservation and NMP.^12-14^ Typically, COR is initiated with a mean arterial pressure of 30 mm Hg at 5 to 10 °C and transitions to 75 mm Hg at 35 to 37 °C during the course of 90 min. Compared with an abrupt rewarming without COR, the implementation of COR has been shown to increase creatinine clearance,^8,13-15^ oxygen utilization efficiency,^8,13^ ATP content^16^ and the amount of ATP produced per milliliter of consumed oxygen,^12^ cortical perfusion,^17^ and mitochondrial recovery^8^ during normothermic reperfusion. Moreover, the introduction of COR has been shown to reduce posttransplant serum creatinine levels,^17^ fractional excretion of sodium (FENa)^8,13,15^ and glucose (FEglu),^13^ urine protein–creatinine ratio,^15^ and the amount of ROS-induced lipid peroxidation^13,17^ during normothermic reperfusion. Although using COR during the course of 90 to 120 min alleviates renal rewarming injury, it remains unclear which rewarming rate is the most protective. Hence, this preclinical study aimed to investigate which rewarming rate of COR results in the least renal rewarming injury during subsequent NMP.

## MATERIALS AND METHODS

### Study Design

**FIGURE 1.**
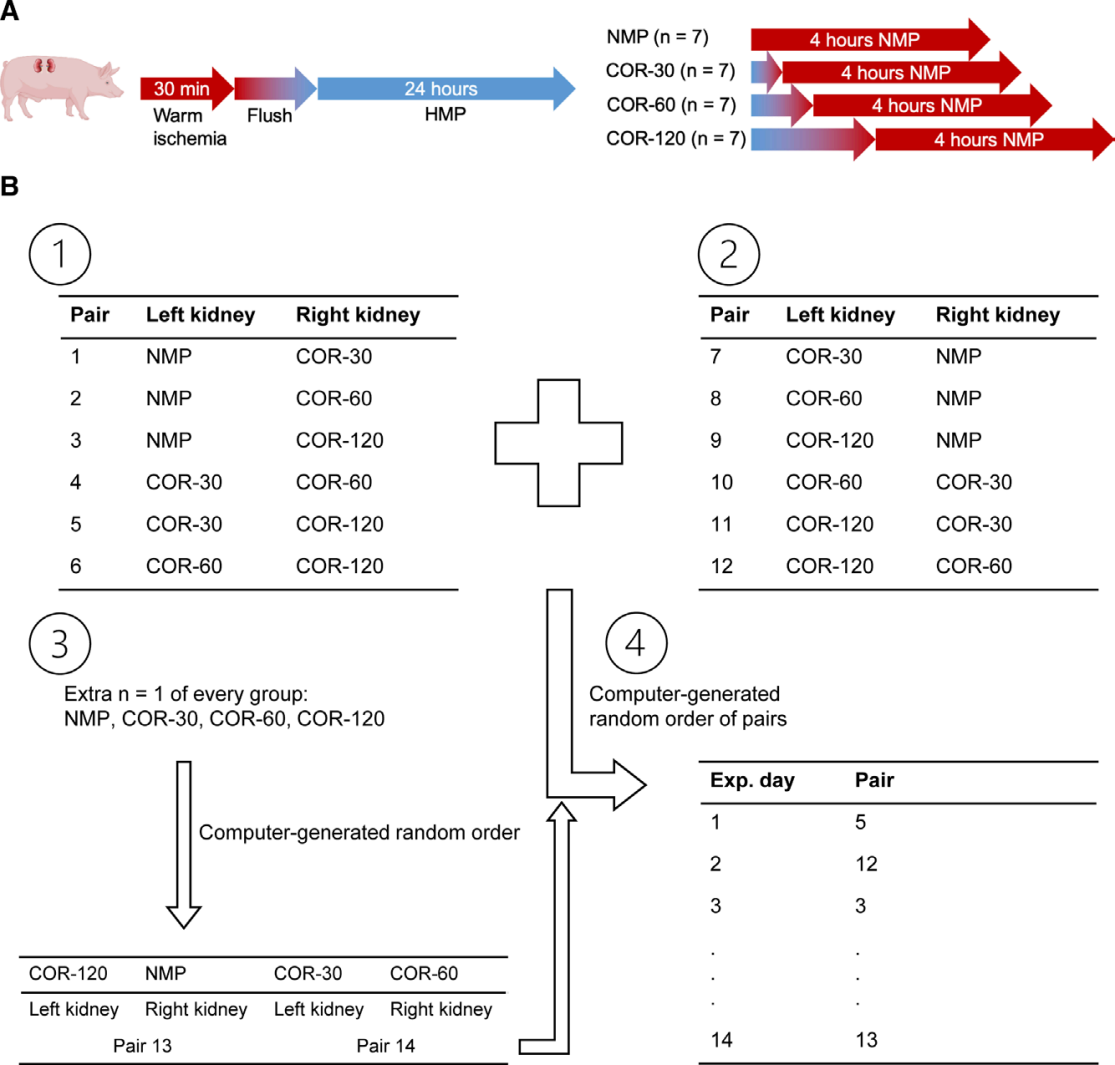
Study design (A) and randomization (B). Following 30 min of warm ischemia, kidneys were flushed with cold saline and subsequently preserved with oxygenated HMP for 24 h. Afterward, kidneys were either rewarmed abruptly by immediately commencing NMP for 4 h or gradually by means of COR during the course of 30, 60, or 120 min before 4 h of NMP. Panel B shows the randomization process of the paired perfusions in which both kidneys from the same pig were simultaneously subjected to (COR-)NMP, using 2 separate circuits. First, each group was paired twice with all other groups, once as the left kidney and once as the right kidney (B1 and B2). Second, to obtain n = 7 per group, all groups were randomly paired with another group 1 more time (B3). Finally, the computer generated a random order of pairs to determine when each pair will be perfused (B4). HMP, hypothermic machine perfusion; COR, controlled oxygenated rewarming; NMP, normothermic machine perfusion.

Twenty-eight porcine kidneys were randomly assigned to 4 groups (n = 7 per group) using computer-generated blocked randomization to be either rewarmed abruptly to 37 °C (NMP group) or gradually from 4–8 °C to 37 °C during the course of 30 min (COR-30 group), 60 min (COR-60 group), or 120 min (COR-120 group; Figure 1A). To minimize interindividual variability, paired perfusions were performed in which both kidneys from the same pig were simultaneously subjected to (COR-)NMP, using 2 separate circuits. The groups that were assigned to the kidneys were determined using computer-generated randomization (Figure 1B).

### Kidney Retrieval

Viable porcine kidneys from domestic landrace pigs (sow; type Topigs 20) and autologous blood were obtained from a local slaughterhouse (Kroon Vlees, Groningen, the Netherlands). The blood was collected in a beaker containing 25 000 international units of heparin (LEO pharma, Ballerup, Denmark). Kidneys underwent approximately 30 min of warm ischemia (WI) while cannulating the renal arteries with a 5-mm straight cannula (Organ Recovery Systems, Itasca, IL) and the ureters with an 8 Ch cannula (Nutrifit Health Ltd, Caterham, United Kingdom). After WI, both kidneys were cold flushed with 500 mL saline (Baxter B.V., Utrecht, the Netherlands) at 4 °C with a hydrostatic pressure of 100 cmH_2_O. Subsequently, the kidneys were connected to an oxygenated hypothermic (1–10 °C) machine perfusion (HMP) device (Kidney Assist Transport, XVIVO, Göteborg, Sweden) and perfused with the University of Wisconsin Machine Perfusion solution (Belzer UW-MP solution, Bridge to Life Ltd, Columbia, SC) for 24 h at a mean arterial pressure of 25 mm Hg. Heparinized autologous whole blood was depleted from leukocytes using a leukocyte filter (BioR 02 plus BS PF, Fresenius Kabi, Zeist, the Netherlands), centrifuged (20 min at 20 °C with 1000*g*), and supernatant plasma was removed, along with supernatant platelets. Next, phosphate-buffered saline was added to the red blood cells (RBCs), after which centrifugation and RBCs isolation were repeated. After this washing step, the RBCs were mixed with phosphate-buffered saline in a 1:1 ratio and stored overnight at 4 °C before they were centrifuged and isolated for the third time. As the kidneys of pigs slaughtered for meat consumption were used, no approval from the ethics committee was required.

### Ex Vivo Machine Perfusion Setup

Before the kidneys were connected to the perfusion circuit, they were flushed with 250 mL of saline at 4 °C with a hydrostatic pressure of 100 cmH_2_O. The machine perfusion setup was similar to previously described setups by our group.^18,19^ The perfusion circuit consisted of a pump unit and centrifugal pump (cirQlife, XVIVO, Göteborg, Sweden), oxygenator (Hilite 800 LT, Medos Medizintechnik AG), LifePort organ chamber (Organ Recovery Systems), and thermal unit (Kidney Assist, XVIVO, Göteborg, Sweden). Pressure was measured at the distal end of the straight cannula (TruWave disposable pressure transducer, Edwards Lifesciences, Irvine, CA), flow was monitored using an ultrasonic clamp-on flow probe (Transonic Systems Europe B.V., Elsloo, the Netherlands), and temperature was monitored using a temperature sensor (Heraeus Nexensos PT100 Surface Sensor [W-SZK], Heraeus Nexensos GmbH, Kleinostheim, Germany), which was positioned underneath the kidney close to the venous outflow. Arterial pressure, perfusate flow, and renal temperature were continuously measured.

### COR and NMP

The perfusion circuit was primed with 245 mL sodium chloride 0.9% (Fresenius Kabi Nederland B.V., Zeist, the Netherlands), 130 mL human albumin 200 g/L (Sanquin Plasma Products B.V., Amsterdam, the Netherlands), 17 mL sodium bicarbonate 8.4% (B. Braun Melsungen AG, Melsungen, Germany), 4 mL calcium gluconate 10% (B. Braun), 7.5 mL mannitol 15% (Baxter B.V., Utrecht, the Netherlands), 10 mL cefuroxime 1500 mg (Fresenius Kabi) dissolved in 20 mL sterile water (Fresenius Kabi), 26 mL Aminoplasmal 10% (B. Braun), 10 mL glucose 5% (Baxter), 0.7 mL magnesium sulfate 100 mg/mL (Teva Nederland B.V., Haarlem, the Netherlands), 0.2 mL sodium phosphate 3 mmol/mL (Apotheek A15, Gorinchem, the Netherlands), 1.8 mL potassium chloride 1 mmol/L (Centrafarm B.V., Etten-Leur, the Netherlands), 75 mL sterile water (Fresenius Kabi), 2 mL verapamil 2.5 mg/mL (Centrafarm B.V.), and 0.0596 g (Sigma-Aldrich, Zwijndrecht, the Netherlands) to achieve an initial circulating concentration of 1000 µmol/L. The perfusate was oxygenated with carbogen (95% O_2_/5% CO_2_) at a rate of 0.5 L/min. Carbogen was preferred over 100% oxygen to ensure an optimal acid–base balance.

**FIGURE 2.**
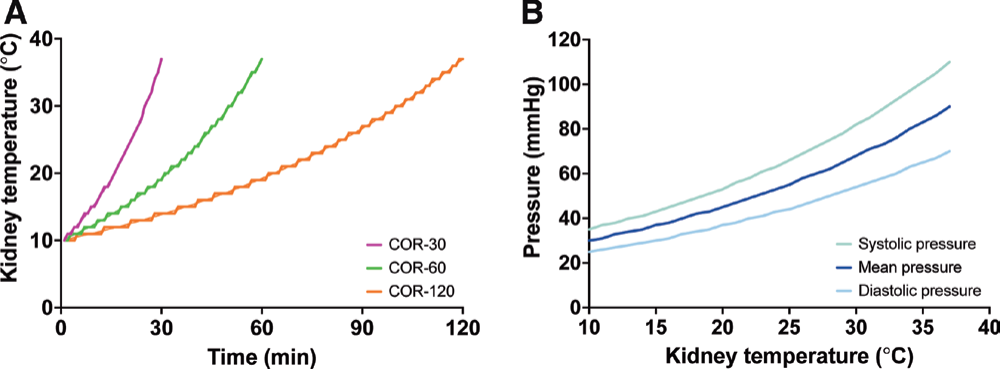
Desired renal rewarming pattern of intervention groups (A) and adapted increase in perfusate pressure (B) during controlled oxygenated rewarming. In panel A, kidney temperature was calculated using the formula $T\left(t\right)=10\times {\left(\frac{37}{10}\right)}^{{\left(\frac{1}{RT}\right)}^{t}}$, in which *T* is the kidney temperature (°C) on time point *t* (in minutes) and RT is the rewarming time (in minutes; either 30, 60, or 120 min).

COR was initiated at conditions similar to HMP (pulsatile pressure of 35/25 mm Hg at 4–8 °C) and gradually transitioned to conditions that resemble NMP (pressure of 110/70 mm Hg at 35–37 °C) during a time span of 30, 60, or 120 min. COR was executed following an exponential temperature pattern (Figure 2). Concomitantly with the increase in temperature, the arterial perfusate pressure was increased. To ensure proper mixing of RBCs with the perfusate, 2 min before the renal temperature was set to 20 °C and 200 mL of perfusate was removed from reservoir and mixed with 250 mL of RBCs. When the temperature was set to 20 °C, this mixture was added to the reservoir to acquire a hematocrit of approximately 32%. After adding the RBCs, the perfusate was supplemented with a mixture of 2.8 mL verapamil 2.5 mg/mL (Centrafarm B.V.), 14.6 mL glucose 5% (Baxter), 70 mL Aminoplasmal 10% (B. Braun), 2 mL Cernevit (Baxter), and 0.35 mL insulin 100 IU/mL (Novo Nordisk A/S, Bagsværd, Denmark) at an infusion rate of 6.2 mL/h. After the perfusion parameters were set to 37 °C with a pressure of 110/70 mm Hg, the kidneys were perfused for 4 h. Whenever arterial glucose concentration reduced to <4.0 mmol/L after the first hour of NMP, a calculated bolus of glucose 5% was administered to the circulating perfusate to achieve a concentration of 6 mmol/L.

### Perfusate, Urine, and Tissue Measurements

Perfusate samples, as well as arterial and venous blood gas samples, were taken 10 min before COR, when the temperature was set to 20 °C, 37 °C (start of NMP), and after 15 min of NMP. Subsequent perfusate and urine samples, as well as additional arterial and venous blood gas samples, were collected hourly. Blood gas analyses enabled the calculation of oxygen consumption (VO_2_) using the following formula^20^:


$$\begin{array}{ll} & VO_{2}\left(mLO_{2}/min\right)\\ & =\left((\left(Hb\times 2.4794\right)+\left(pO_{2}arterial\times K\right)\right)\\ & -\left(\left(0.024794\times Hb\times SO_{2}venous\right)+\left(pO_{2}venous\times K\right)\right))\times Q \end{array}$$

where Hb is the hemoglobin concentration (mmol/L), pO_2_ is the partial oxygen pressure (kPa), K is the solubility constant of oxygen in water at 37 °C that equals 0.0225 (mL O_2_ per kPa), SO_2_ is the saturation (%), and Q is the renal perfusate flow (dL/min).

Perfusate and urine concentrations of creatinine, sodium, and glucose were determined in our hospital’s clinical laboratory by using routine clinical assays. The addition of creatinine to the perfusate enabled the measurement of renal function during NMP. Creatinine clearance and the fractional excretion of sodium and glucose were calculated hourly.

The fractional excretion of sodium and glucose was calculated using the following formula:

$FENa/glu\left(\%\right)\;=\frac{urine[Na/glu]\times perfusate[creatinine]}{(urine\left[creatinine\right]\times perfusate[Na/glu])}\times 100$

The efficiency of renal oxygen utilization was calculated by dividing the total sodium transport (TNa) by VO_2_ (in mmol O_2_/min/100*g*) using the following formula:

$TNa\left(mmolNa^{+}/mmolO_{2}\right)\;=\frac{\left(\left(Crclearance\times perfusate\left[Na\right]\right)-\left(urineflow\times urine\left[Na\right]\right)\right)}{VO_{2}}$

Cortical punch biopsies (4 mm) were obtained before the start of normothermic perfusion, when the perfusate temperature reached 37 °C (in COR groups), after 120 min of NMP, and after 240 min of NMP. All biopsies were vertically transected into 2 equally large pieces, one of which was snap-frozen, and the other half was stored in sonification solution (SONOP; containing 0.372 g EDTA in 130 mL H_2_O and NaOH [pH of 10.9] and 370 mL ethanol 96%, as previously described^20^). After obtaining the cortical biopsies, they were stored in liquid nitrogen during the experiments and subsequently preserved at –80 °C.

Assessment of renal injury was performed on cortical snap-frozen biopsies taken at 240 min NMP by measuring the tissue expression of 4-hydroxy-2-nonenal (HNE) as an indicator of ROS-induced cellular injury. HNE was measured using a porcine-specific ELISA kit (Porcine 4 Hydroxynonenal ELISA kit, BlueGene Biotech, Shanghai, China) according to manufacturers’ protocol. Tissue ATP levels were measured in the cortical tissue SONOP samples at all time points as an indication of energetic recovery. ATP levels were determined with an ATP Bioluminescence assay kit CLS II (Boehringer, Mannheim, Germany) and a luminometer (Victor^3^ 1420 multilabel counter, PerkinElmer) using a standard protocol as previously described by our group^21^ and expressed in micromole per gram protein.

### Statistical Analysis

GraphPad Prism version 9.3.1 (GraphPad Software Inc., La Jolla, CA) was used to perform statistical analyses. The normality of the data was analyzed with the Shapiro-Wilk test and the homogeneity of variances was tested with Bartlett test. If data were normally distributed and if standard deviations (SDs) were not significantly different (*P* ≤ 0.05), Tukey’s multiple comparison test was used. In case of normally distributed data with significantly different SDs, a 1-way analysis of variance with Dunnett’s T3 multiple comparisons test was used. Otherwise, the Kruskal–Wallis test with Dunn’s multiple comparisons test was used. Continuous data are presented as means (SD) and a 2-sided *P* ≤ 0.05 was considered to indicate statistical significance.

## RESULTS

Twenty-eight porcine kidneys (n = 7 per group) were included. There were no significant differences in WI time, HMP time, renal weight, and weight increase between the groups.

### Thermal Transition Characteristics

**FIGURE 3.**
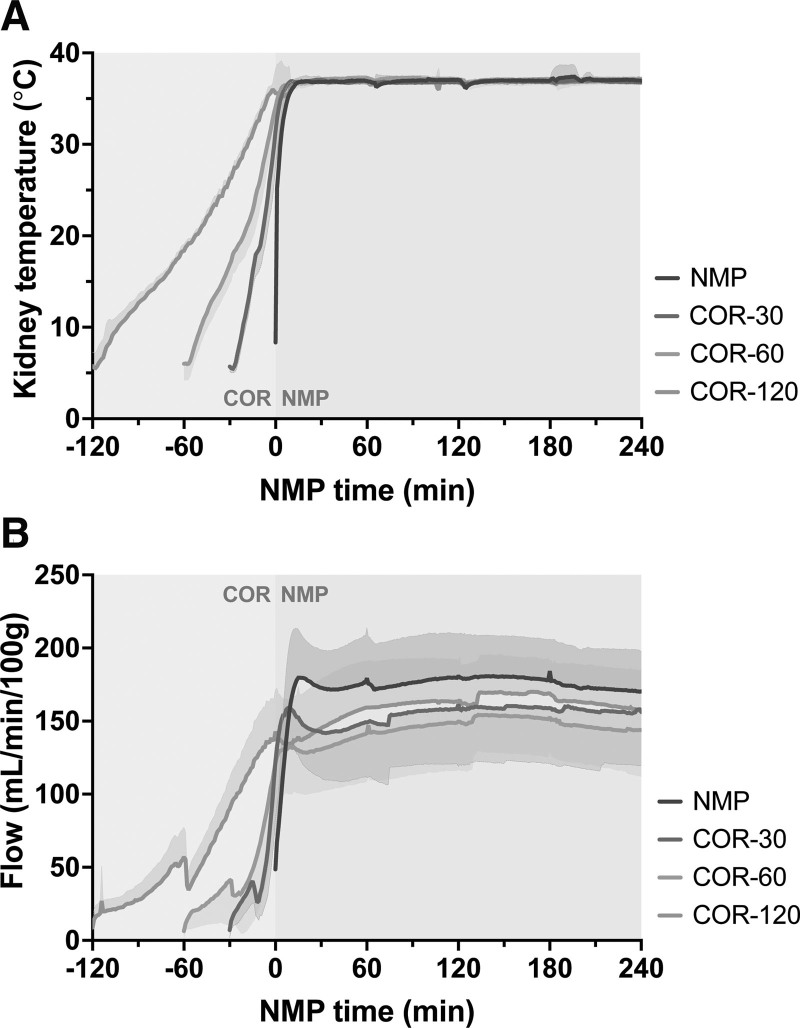
Kidney temperature (A) and perfusate flow (B) during COR and NMP. Means and standard deviations are shown. COR, controlled oxygenated rewarming; COR-30, -60, or -120, controlled oxygenated rewarming during the course of 30, 60, or 120 min; NMP, normothermic machine perfusion.

After 30 min (±10%) of WI and 24 h of HMP, the kidneys were rewarmed either abruptly (NMP) or gradually during the course of 30, 60, or 120 min (Figure 3). After normothermic temperatures were reached, the increase in renal perfusate flow flattened and remained relatively constant during the course of NMP. In the NMP group, perfusate flow increased sharply and reached a plateau within the first 15 to 30 min of perfusion. Renal perfusate flow during 4 h of NMP was significantly higher in the NMP group compared with all COR groups (*P* < 0.0001 for all comparisons), in the COR-120 compared with the COR-30 (*P* < 0.0001) and COR-60 groups (*P* < 0.0001), and in the COR-30 group compared with the COR-60 group (*P* < 0.0001; NMP, 172.9 ± 15.94 mL/min/100 g; COR-30, 154.4 ± 6.17 mL/min/100 g; COR-60, 144.2 ± 7.67 mL/min/100 g; COR-120, 159.3 ± 9.43 mL/min/100 g).

### Functional Parameters

**FIGURE 4.**
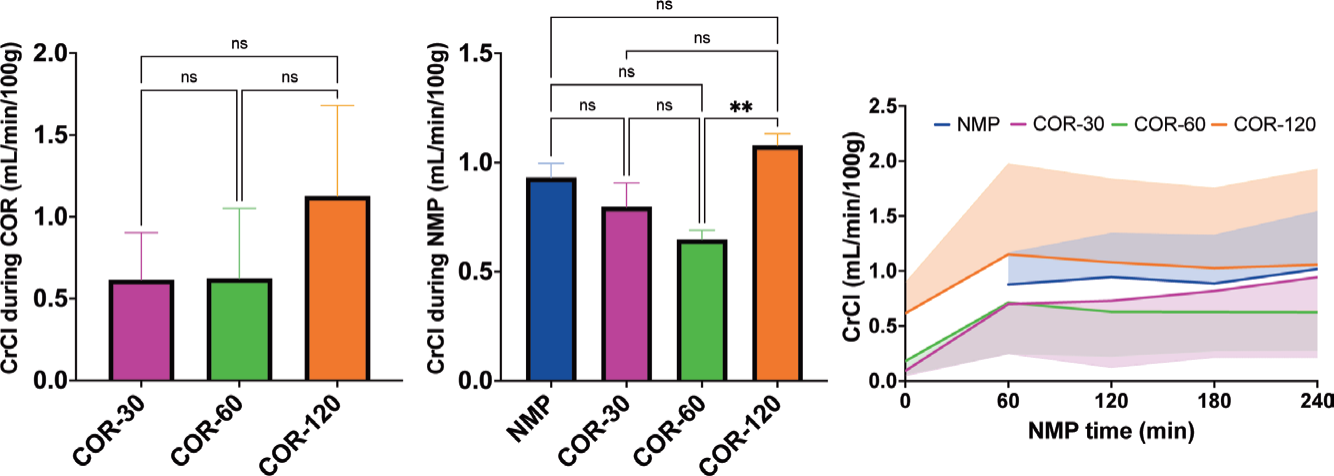
Creatinine clearance during COR and NMP. Means and standard deviations are shown. COR, controlled oxygenated rewarming; COR-30, -60, or -120, controlled oxygenated rewarming during the course of 30, 60, or 120 min; CrCl, creatinine clearance; NMP, normothermic machine perfusion. ^**^P < 0.01.

Creatinine clearance was calculated to serve as a surrogate marker for the glomerular filtration rate. In all COR groups, creatinine clearance (CrCl) was relatively low during rewarming and increased rapidly during the first hour of NMP (Figure 4). After the initial increase in the COR groups upon normothermic perfusion, the CrCl remained almost constant during NMP. Over the course of 4 h NMP, the mean CrCl was significantly higher in COR-120 compared with COR-60 (1.08 ± 0.05 versus 0.65 ± 0.04 mL/min/100 g, *P* < 0.01).

**FIGURE 5.**
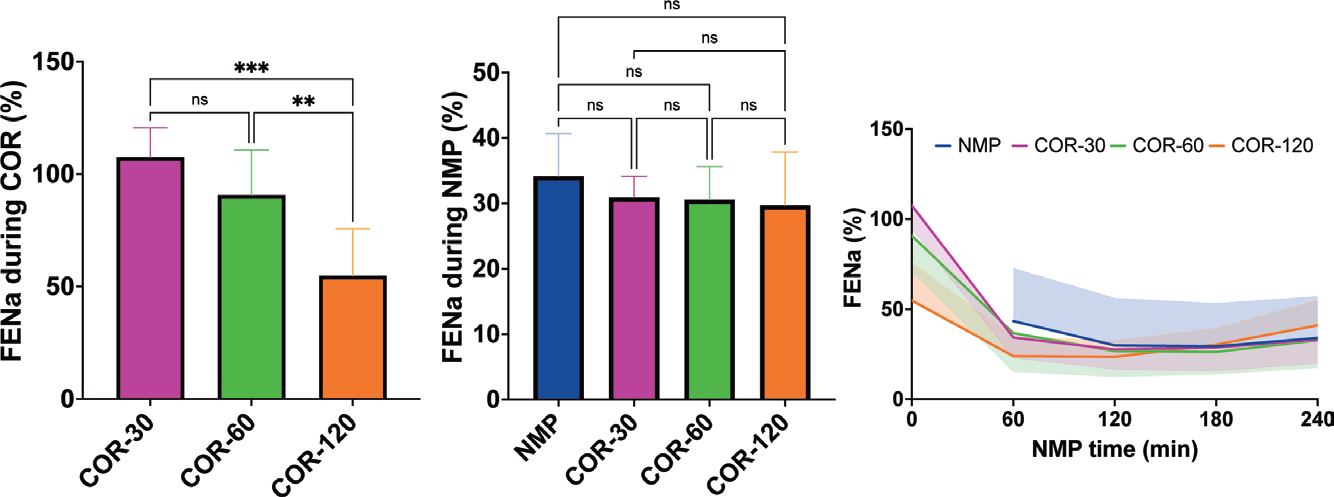
Fractional excretion of sodium during COR and subsequent NMP. Means and standard deviations are shown. COR-30, -60, or -120, controlled oxygenated rewarming during the course of 30, 60, or 120 min; FENa, fractional excretion of sodium; NMP, normothermic machine perfusion. ^**^P < 0.01, ^***^P < 0.001.

**FIGURE 6.**
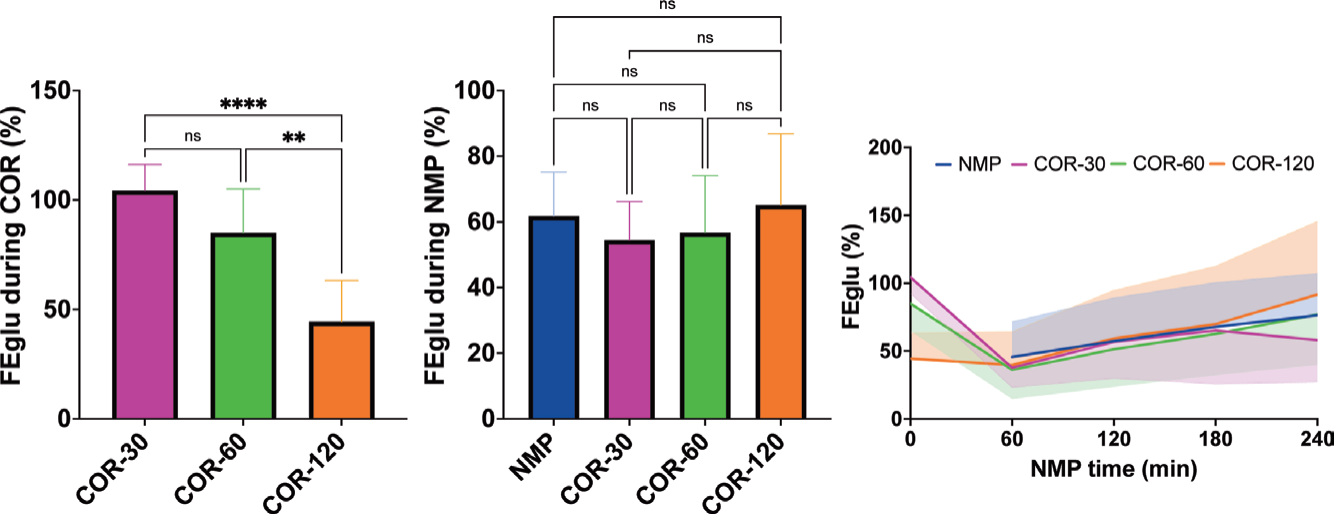
Fractional excretion of glucose during COR and subsequent NMP. Means and standard deviations are shown. COR-30, -60, or -120, controlled oxygenated rewarming during the course of 30, 60, or 120 min; FEglu, fractional excretion of glucose; NMP, normothermic machine perfusion. ^**^P < 0.01, ^****^P < 0.0001.

To evaluate tubular transport function, FENa and FEglu were calculated. In contrast to CrCl, FENa was relatively high during COR and rapidly decreased during the first hour of NMP (Figure 5). A more gradual rewarming during the course of 120 min resulted in significantly lower FENa (54.81 ± 20.85%) compared with a rewarming duration of 30 (107.5 ± 13.04%, *P* < 0.001) and 60 min (90.71 ± 19.96%, *P* < 0.01) upon NMP. However, this difference disappeared during subsequent NMP. Similarly, in the COR-30 and COR-60 groups, FEglu was relatively high during COR and decreased rapidly during the first hour of NMP, after which it gradually increased (Figure 6). However, in the COR-120 group, the FEglu (44.47 ± 18.78%) was significantly lower during COR compared with the COR-30 (104.3 ± 11.85%, *P* < 0.0001) and COR-60 groups (84.99 ± 20.04%, *P* < 0.01). In addition, in the COR-120 group, FEglu remained relatively constant during the first hour of NMP, after which it gradually increased. The NMP group also showed an increase in FEglu over time.

### Oxygen Utilization Efficiency, Energetic Recovery, and ROS-induced Injury

**FIGURE 7.**
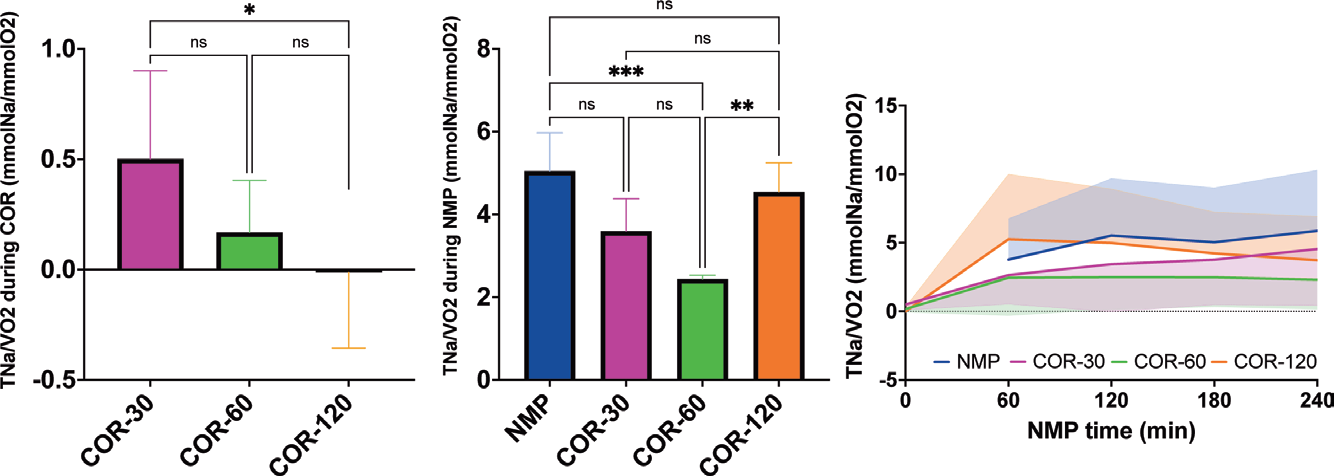
Oxygen utilization efficiency during COR and subsequent NMP. Means and standard deviations are shown. COR-30, -60, or -120, controlled oxygenated rewarming during the course of 30, 60, or 120 min; NMP, normothermic machine perfusion; TNa, total sodium transport; VO_2_, oxygen consumption. ^*^P < 0.05, ^**^P < 0.01, ^***^P < 0.001.

Renal oxygen utilization efficiency was approximated as the ratio of TNa to VO_2_. The efficiency of oxygen utilization by the kidney tissue was relatively low during COR and increased during the first hour of NMP (Figure 7). Moreover, the oxygen utilization efficiency was significantly lower in the COR-120 group (–0.01 ± 0.34 mmolNa/mLO_2_) compared with the COR-30 group (0.50 ± 0.40 mmolNa/mLO_2_, *P* < 0.05) during COR. However, this significance disappeared during NMP. In fact, the oxygen utilization efficiency was numerically higher in the COR-120 group (4.55 ± 3.62 mmolNa/mLO_2_) compared with the COR-30 group (3.60 ± 3.21 mmolNa/mLO_2_) during NMP. Moreover, during NMP, both COR-120 and NMP (5.05 ± 3.78 mmolNa/mLO_2_) groups showed a significantly more efficient oxygen utilization compared with the COR-60 group (2.44 ± 2.24; *P* < 0.001 versus NMP, *P <* 0.01 versus COR-120).

**FIGURE 8.**
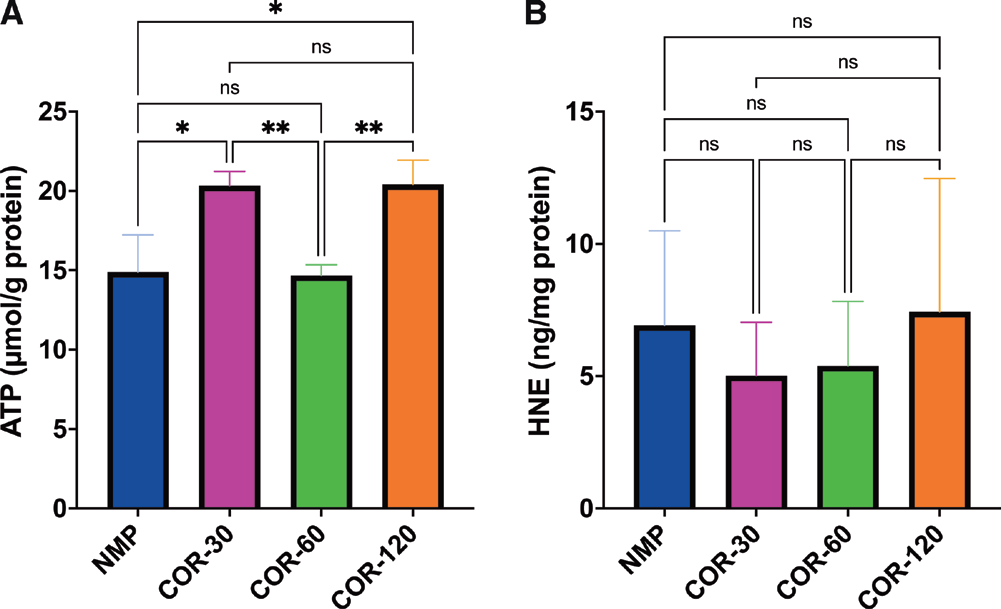
ATP (A) and 4-hydroxy-2-nonenal (B) levels during normothermic perfusion. Means and standard deviations are shown. ATP, adenosine triphosphate; COR-30, -60, or -120, controlled oxygenated rewarming during the course of 30, 60, or 120 min; HNE, 4-hydroxy-2-nonenal; NMP, normothermic machine perfusion. ^*^P < 0.05, ^**^P < 0.01.

Energetic recovery was approximated by the tissue ATP concentration. Over the course of NMP, the tissue concentration of ATP was significantly higher in the COR-120 (20.41 ± 5.95 µmol/g protein) as well as the COR-30 group (20.32 ± 8.51 µmol/g protein) compared with the COR-60 (14.67 ± 3.96 µmol/g protein; *P* < 0.01 for both comparisons) and NMP groups (14.89 ± 5.58 µmol/g protein; *P* < 0.05 for both comparisons; Figure 8). The degree of ROS-induced cellular injury was expressed as tissue concentration of HNE. As cellular injury through ROS-induced lipid peroxidation accumulates during perfusion, HNE levels were measured at the end of normothermic perfusion (ie, after 4 h of NMP). Overall, the HNE concentration was numerically higher in the NMP (6.92 ± 3.57 ng/mg protein) and COR-120 (7.43 ± 5.04 ng/mg protein) groups compared with the COR-30 (5.01 ± 2.02 ng/mg protein) and COR-60 (5.38 ± 2.45 ng/mg protein) groups. However, these differences did not reach statistical significance.

## DISCUSSION

To the best of our knowledge, this is the first study to address the relationship between the degree of rewarming during COR and the degree of rewarming injury during reperfusion when COR is implemented as a thermal transition phase between HMP and NMP. Our data showed that after COR (ie, at the start of NMP), tubular function is better preserved when COR consists of a lower rewarming rate (more gradual rewarming). However, this significance disappeared during the course of NMP. In contrast to this finding, we showed that during NMP, the utilization of oxygen was most efficient in the kidneys that were rewarmed more gradually (120 min COR) compared with those that were rewarmed less gradually (60 min COR). Energetic recovery is of utmost importance for renal graft function. It has been suggested that the amount of ATP, thereby the energetic recovery, reflects mitochondrial health.^22^ A less gradual (30 min COR) and more gradual (120 min COR) rewarming resulted in higher ATP levels during NMP than a medium-gradual (60 min COR) and an abrupt rewarming pattern. Interestingly, the degree of rewarming during COR did not show any association with the degree of ROS-induced cellular injury through lipid peroxidation.

Whether or not a more gradual rewarming can result in additional renal protection and thereby prevent deterioration of renal function because of an abrupt thermal transition from hypothermia to normothermia depends on parameters that reflect renal viability and rewarming injury. Mitochondrial dysfunction is thought to be the main culprit of rewarming injury. Hence, parameters that reflect mitochondrial health are crucial in determining the degree of rewarming injury. One such parameter is ATP concentration. It has been shown that regeneration of ATP during reperfusion is closely related to tissue viability and survival rate of the animal.^23,24^ Moreover, Leducq et al^11^ demonstrated a rapid decline in ATP content of perfused organs during the hypothermic to normothermic transition. To elucidate whether more gradual rewarming can alleviate the decline in ATP content, Hoyer et al^25^ and Minor et al^16^ investigated the difference in regeneration of ATP during reperfusion of human livers when either gradual or abrupt rewarming preceded it. They described superior energetic recovery when gradual instead of abrupt rewarming was implemented before reperfusion. Hence, the reduction in tissue ATP content and the concomitant reduction in tissue viability because of an abrupt thermal transition from hypothermia to normothermia can be alleviated by gradually rewarming the organ. Our findings of improved ATP levels when kidneys were rewarmed during the course of 30 or 120 min during COR compared with abrupt rewarming are in line with these results and confirm that COR results in superior preservation of tissue viability. Accordingly, Zlatev et al^8^ showed that COR improves mitochondrial spare respiratory capacity, oxidative phosphorylation coupling efficiency, and aerobic efficiency during reperfusion. Moreover, Minor and von Horn^12^ postulated that the efficiency of ATP synthesis (expressed as the amount of tissue ATP per mL of consumed oxygen) is improved by gradual rewarming.

Although under physiological conditions, most of the consumed oxygen is used by the kidney to produce ATP, reoxygenation during reperfusion results in excessive ROS production.^9^ ROS, in turn, can induce damage to adjacent biomolecules such as lipids, which results in lipid peroxidation.^10^ It has been shown that the amount of lipid peroxidation is increased during abrupt rewarming.^26,27^ Also, a study by von Horn et al^13^ showed that the concentration of lipid peroxidation products thiobarbituric acid-reactive substances and HNE are significantly reduced during reperfusion when 90 min of COR and 30 min of NMP (total of 2-h perfusion) preceded simulated reperfusion compared with NMP alone. In a porcine autotransplantation study, Gallinat et al^17^ drew similar conclusions, showing that the plasma concentration of thiobarbituric acid–reactive substances was significantly reduced during the first week after transplantation when kidneys were subjected to COR before implantation, compared with kidneys that were preserved by means of static cold storage without gradual rewarming. In contradiction to earlier findings, our experiments did not show meaningful reduction in HNE during the course of perfusion when COR was implemented before NMP. Interestingly, in contrast to earlier work on COR from a group from Essen, Germany,^8,13-15^ implementation of COR did not lead to improvement of creatinine clearance during NMP. Although our data showed that immediate renal tubular function, characterized by lower fractional excretion of sodium and glucose, was significantly enhanced with longer COR duration (lower rewarming rates) upon initiation of NMP, this enhancement became nonsignificant over the course of normothermic perfusion.

In contrast to the experiments of von Horn et al,^13^ our data did not show a significantly different TNa/VO_2_ between COR and abrupt rewarming (NMP without COR), nor did our experiments show consistent significant differences in glomerular (creatinine clearance) and tubular function (fractional excretion of sodium and glucose). A possible explanation for this is that our experiments did not incorporate a reperfusion model; as a result, it remains unclear whether larger differences would become visible after reperfusion. Hence, it remains to be demonstrated whether our observations translate into improved renal function after transplantation. In addition, the use of slaughterhouse kidneys might have resulted in higher interindividual variability in kidney viability and extent of renal injury, which could interfere with our outcome measurements. Moreover, it is possible that significant differences could not be reached during the relatively short 4 h of NMP. Another limitation of this study is that we did not incorporate every aspect of rewarming injury in our assays. Our analysis did not include vascular injury or apoptosis. Nevertheless, vascular injury and apoptosis would also affect glomerular and tubular function; therefore, they are indirectly incorporated when assessing renal function. In addition, it takes time for some of the underlying processes of rewarming injury to become apparent, making them less suitable for relatively short assessment periods during NMP. Furthermore, our experiments did not have a second cold ischemic period, as would be observed during implantation when vascular anastomosis is performed. However, this study aimed to investigate the effect of rewarming injury during normothermic perfusion when COR was implemented as a thermal transition phase between 2 different machine perfusion modalities (HMP-COR-NMP), thereby providing the first evidence that the gradualness of COR affects renal function during normothermic perfusion.

In conclusion, this study suggests that a rewarming duration of 120 min with a rewarming rate of 1.10%/min (on average 0.225 °C/min) during COR could result in superior immediate tubular function and energetic recovery during subsequent NMP. Therefore, our data suggest that a rewarming rate of 1.10%/min during the course of 120 min may provide the best protective effect against renal rewarming injury during subsequent NMP. Although this project showed that energetic recovery is enhanced with gradual rewarming compared with abrupt rewarming, gradual rewarming did not result in superior protection to other aspects of rewarming injury. Hence, future studies that incorporate kidney transplantation should compare the posttransplant outcomes of gradually rewarmed donor kidneys with abruptly rewarmed renal grafts.

## ACKNOWLEDGMENTS

The authors would like to thank Petra Ottens for her help with NMP procedures and analyses.
